# Télangiectasies digestives révélant une sarcoïdose systémique

**DOI:** 10.11604/pamj.2015.22.101.8005

**Published:** 2015-10-06

**Authors:** Madiha Mahfoudhi, Khaled Khamassi

**Affiliations:** 1 Service de Médecine Interne A, Hôpital Charles Nicolle, Tunis, Tunisie; 2 Service ORL, Hôpital Charles Nicolle, Tunis, Tunisie

**Keywords:** Télagiectasie, fibroscopie digestive, biopsie, sarcoïdose, telangiectasia, gastroscopy, biopsy, sarcoidosis

## Image en medicine

La sarcoïdose systémique est une maladie granulomateuse qui atteint avec prédilection l'appareil respiratoire. Une granulomatose digestive étagée isolée est rarement révélatrice de cette maladie. Le résultat de l'examen histologique de plusieurs biopsies étagées est déterminant. Patient âgé de 47 ans sans antécédents particuliers a consulté pour des épigastralgies atypiques évoluant depuis 6 mois non améliorées par traitement symptomatique. Il n'avait pas de fièvre, ni d'altération de l’état général, ni de xérostomie. L'examen physique en particulier abdominal et respiratoire était sans anomalies. L'examen biologique ainsi que la radiographie de thorax étaient normaux. Le Quantiféron TB test ainsi que l'intradermo-réaction à la tuberculine était négatifs. La fibroscopie digestive haute a révélé des télangiectasies ‘sophagiennes, gastriques et duodénales. L'examen histologique de biopsies digestives étagées a conclut à une granulomatose digestive étagée. Il s'agissait de granulomes giganto-cellulaires sans nécrose caséeuse. Plusieurs diagnostics ont été suspectés notamment une tuberculose, un lymphome et une sarcoïdose. La recherche de BK dans les crachats et les urines par un examen direct et des cultures était négative. Le dosage de l'enzyme de conversion de l'angiotensine était à 2 fois la normale. Une fibroscopie bronchique avec biopsies étagées et étude du lavage broncho-alvéolaire étaient sans anomalies. L’étude d'une biopsie des glandes salivaires accessoires a montré aussi des lésions de granulomes giganto-cellulaires sans nécrose caséeuse. Le diagnostic d'une sarcoïdose systémique à localisation digestive et salivaire a été retenu. Le traitement s'est basé sur une corticothérapie. L’évolution était favorable sur le plan clinique et endoscopique.

**Figure 1:**
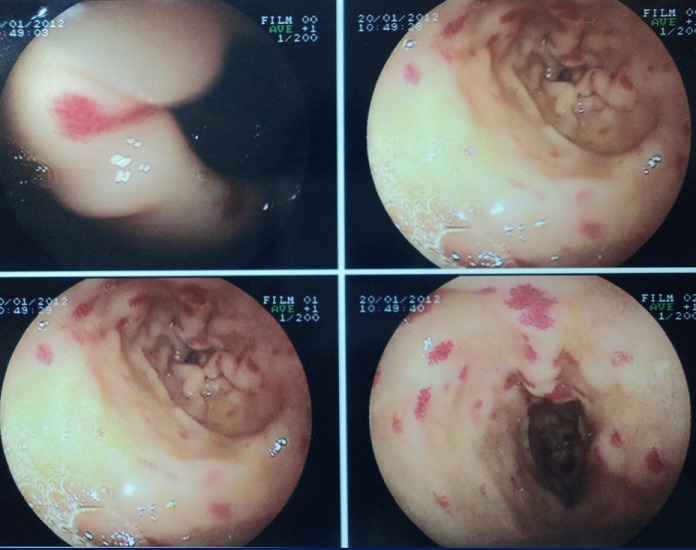
Télangiectasies œsophagiennes, gastriques et duodénales

